# Tongmai Yangxin intervening in myocardial remodeling after PCI for coronary heart disease: study protocol for a double-blind, randomized controlled trial

**DOI:** 10.1186/s13063-020-4208-4

**Published:** 2020-03-20

**Authors:** Yongxia Wang, Xinlu Wang, Jianru Wang, Bin Li, Rui Yu, Yucai Hu, Xingyuan Li, Guangcao Peng, Mengmeng Zhang, Mingjun Zhu

**Affiliations:** 1 Center of Cardiology, The First Affiliated Hospital of Henan University of TCM, 19 Renmin Road, Jinshui District, Zhengzhou, 450100 Henan Province China; 2 Graduate Division, The First Affiliated Hospital of Henan University of TCM, 19 Renmin Road, Jinshui District, Zhengzhou, 450100 Henan Province China; 3 Henan University of Chinese Medicine, 156 Jinshui East Road, Zhengdong New District, Zhengzhou, 450046 Henan Province China

**Keywords:** Traditional Chinese medicine, Tongmai Yangxin pill, Myocardial remodeling, Acute myocardial infarction

## Abstract

**Background:**

Coronary heart disease (CHD) has become a common cardiovascular disease that seriously threatens the health of people. As reperfusion in the early phase and drug therapy, especially percutaneous coronary intervention (PCI), have become widely used in the clinic, the mortality of acute myocardial infarction in the short term has been reduced significantly. In addition, in 40%–56% of patients who experience myocardial infarction, cardiac dysfunction occurs and about 25%–33% develop heart failure.

**Methods:**

This study was designed as a multicenter, double-blind, randomized, placebo-controlled, parallel-group, superiority trial. Participants were randomly assigned in a 1:1 ratio through a centrally controlled, computer-generated, simple randomization schedule. The primary outcome was left ventricular end-diastolic volume index = left ventricular end-diastolic volume/body surface area. The combined secondary outcomes include traditional Chinese medicine syndrome score, echocardiogram results, 6-minute walk test results, Seattle Angina Questionnaire score, cardiac magnetic resonance imaging results, biological indicators, dynamic electrocardiogram results, and experiment event rate. Assessments will be performed at baseline and at 4, 8, and 12 weeks after randomization.

**Discussion:**

This trial will demonstrate that the addition of a Tongmai Yangxin pill (TMYX) to conventional treatment will intervene in the development of cardiac remodeling and cardiac dysfunction.

**Trial registration:**

This study was registered in the Chinese Clinical Trial Registry on 7 May 2019. The registration number is ChiCRT1900023023 (http://www.chictr.org.cn/showproj.aspx?proj=12370).

## Introduction

Coronary heart disease (CHD) has become a common cardiovascular disease that seriously threatens the health of people. The data from the “Summary of the 2018 Report on Cardiovascular Diseases in China” showed that the number of individuals with cardiovascular diseases has reached 290 million, 11 million of whom have CHD, and 2.5 million have had a myocardial infarction (MI) [1]. As reperfusion in the early phase and drug therapy, especially percutaneous coronary intervention (PCI), have been widely used in the clinic, the mortality of acute MI (AMI) in the short term is reduced significantly. However, unluckily, an epidemiological investigation showed that, although the mortality of CHD has declined, the incidence of heart failure (HF) has increased year by year [2]. Over the past three decades, preventive and therapeutic agents have substantially improved the prognosis of AMI by normatively using β-blockers, angiotensin-converting enzyme inhibitors/angiotensin II receptor antagonists (ACEIs/ARBs), aldosterone receptor antagonists, and so on. However, a report showed that 40%–56% of the patients who experience MI will experience cardiac dysfunction and that about 25%–33% will develop HF [3, 4]. Cardiac remodeling is the key pathophysiological mechanism of angina pectoris, arrhythmia, and HF following MI.

Traditional Chinese medicine (TCM) has been used for thousands of years to treat CHD, as it is characterized by multiple components, and it can intervene and treat diseases through multiple pathways and targets. A number of clinical trials have demonstrated the unique advantage of TCM in the improvement of the clinical symptoms and evolution and prognosis of diseases [5]. However, effective herbal medicine with a high level of evidence is lacking for interventions in ventricular remodeling.

TMYX, formerly known as pill 651, which is composed of the classic formulas “Zhigancao Decoction” and “Sheng mai yin”, has been on the market since 1965 and is registered in the 2015 Chinese Pharmacopoeia. TMYX has been used for decades to cure CHD, chest pain, palpitation, angina, and irregular heartbeats. TMYX consists of 11 Chinese medicinal herbs: Radix Rehmanniae, Caulis Spatholobi, Radix Glycyrrhizae, Ramulus Cinnamomi, Radix Ophiopogonis, Radix Polygoni multiflori preparata, Asini Corii colla, Fructus Schisandrae, Radix Codonopsis, Capapax et Plastrum Testudinis, and Fructus Jujubae. A recent study identified 80 compounds from a TMYX, including flavonoids, coumarins, iridoid glycosides, saponins, and lignans, exhibiting anti-inflammatory activity [6]. A clinical study by Xuemeng Cai et al. [7] indicated that TMYX in the treatment of stable coronary artery disease may be involved in energy metabolism, amino acid metabolism, oxidative stress, and inflammation.

Therefore, TMYX is worth investigating in patients who have experienced AMI and who received PCI because of pleiotropic effects. The assumption is made that TMYX in addition to standardized Western medications may be superior to intervene in myocardial remodeling, decrease the rate of cardiac dysfunction, and delay the occurrence of HF.

## Methods/design

### Study objectives

The study objective is to prove the hypothesis that TMYXP in addition to standardized Western treatment is more effective than standardized Western treatment alone in reducing left ventricular end-diastolic volume index (LVEDVI), intervening in myocardial remodeling after PCI for AMI.

### Design overview

**Fig. 1:**
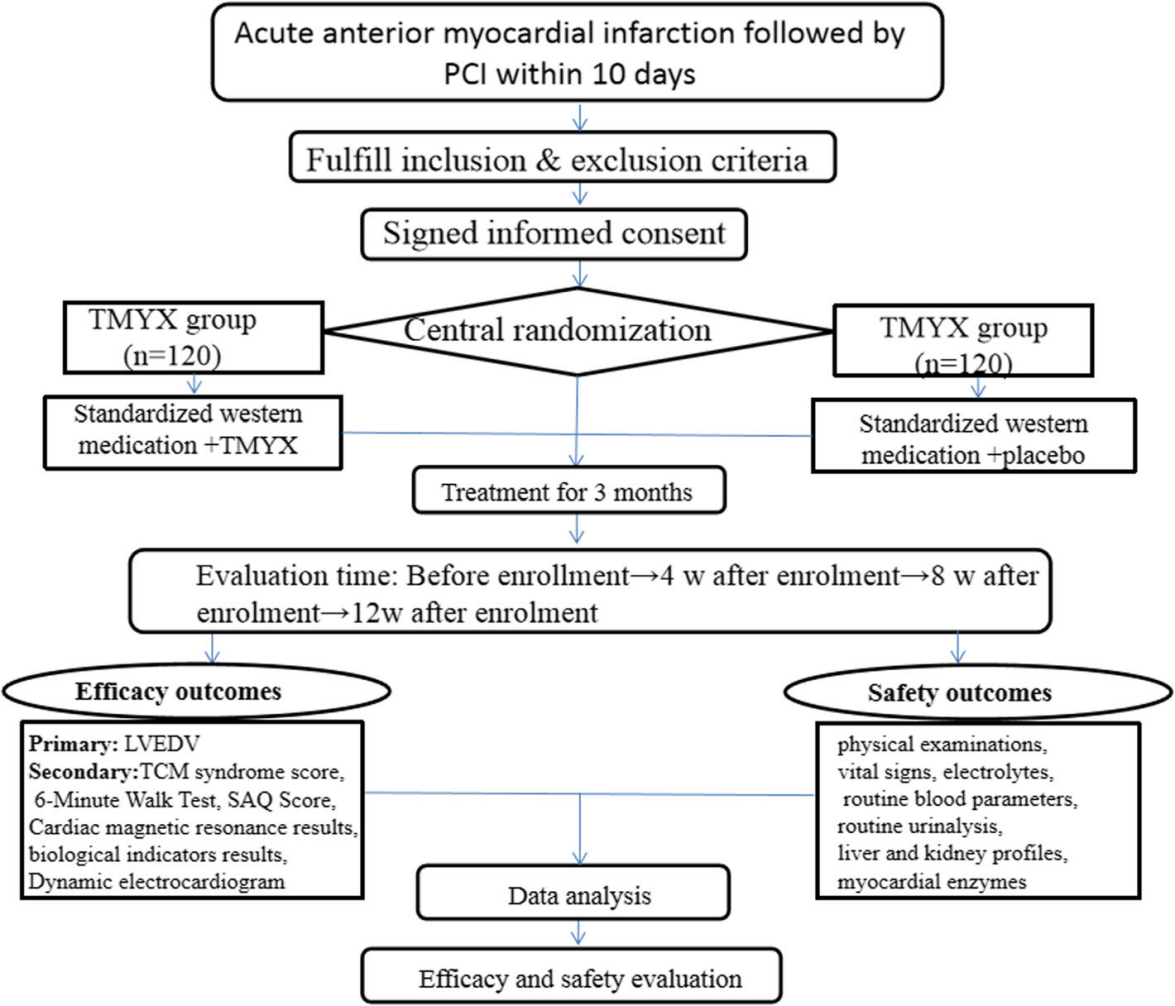
Study design of the Tongmai Yangxin pill (TMYX) trial

This study was designed as a multicenter, double-blind, randomized, placebo-controlled, parallel-group, superiority trial. Eight clinical centers from different regions of China will participate in this trial, including the First Affiliated Hospital of Henan University of Chinese Medicine, the First Affiliated Hospital of Guangzhou University of TCM, Guangdong Provincial Hospital of Traditional Chinese Medicine, the Second Affiliated Hospital of Tianjin University of Traditional Chinese Medicine, Guang’anmen Hospital of the China Academy of Traditional Chinese Medicine, Shuguang Hospital Affiliated to Shanghai University of Traditional Chinese Medicine, and the First Affiliated Hospital of Xinxiang Hospital. All of the researchers of the participating organizations are responsible for data collection and quality. Data management and statistical analyses will be performed solely by data handlers and data analysts at the Institute of Basic Research in Clinical Medicine, China Academy of Chinese Medical Sciences. This study will abide by the Declaration of Helsinki and has been subjected to vetting by the ethics committee of the First Affiliated Hospital of Henan University of TCM (ethics approval number: 2019HL-048-01). As of October 10, 2019, this study was approved by the ethics committees of seven centers (ethics approval numbers: 2019HL-048-01, 2019–095-KY-04, K[2019]041, 2019–693–48-01, 2019–091-01, 2019164, and BF2019–085-01). We will not begin recruiting at other centers in the trial until local ethical approval has been obtained. This study has also been registered with the Chinese Clinical Trial Registry (ChiCRT1900023023). All study subjects must sign and date informed consent documents prior to randomization. A flow diagram of the study procedures is illustrated in Fig. 1.

### Recruitment

We will set up recruitment advertisements to ensure that the subjects are recruited on time. Participants will be recruited through Internet advertisements and posters in the community and at selected hospitals until the whole trial is complete. Jianru Wang, who participated in the revision of the manuscript, will manage the responses to the advertisements and posters, and the researchers who have a Good Clinical Practice certificate will recruit patients.

### Inclusion criteria

This trial will recruit participants who satisfy the following conditions:

1. Patients with acute anterior MI who, within 10 days, underwent emergency PCI, underwent PCI after emergency thrombolytic therapy, or received elective PCI.
2. Patients who meet the syndrome differentiation standard of TCM with combined deficient Qi and Yin blood stasis.
3. Patients who are 18 to 75 years old, both male and female.
4. Patients who voluntarily sign informed consent forms.

### Exclusion criteria

This trial will exclude patients who meet any one of the following criteria:

1. Patients with multiple coronary artery branch lesions who did not receive elective PCI within 10 days.
2. Patients with AMI with severe complications, such as acute HF, cardiogenic shock (conventional treatment cannot correct), or mechanical complications.
3. Patients with AMI with HF, Killip classification III or IV (Killip classification for AMI:
  Class I: no symptoms or signs;
  Class II: has symptoms and signs, rales of not more than 50%, S3 gallop, x-ray pulmonary congestion;
  Class III: severe pulmonary edema, rales of at least 50%;
  Class IV: cardiac shock.)
4. Patients with chronic HF or previous anterior MI in their medical history.
5. Patients with other diseases, such as cardiomyopathy, rheumatic heart disease, valvular heart disease (severe stenosis/incomplete closure), arrhythmia (persistent atrial fibrillation/atrial flutter, third-degree A-V block, sick sinus syndrome, or arrhythmia after the implantation of a pacemaker), hyperthyroidism, cardiac tamponade, pulmonary arterial hypertension, acute paroxysmal asthma/chronic obstructive pulmonary disease, or severe infection.
6. Patients with severe liver and kidney function impairment (alanine aminotransferase (ALT), aspartate transaminase (AST), or total bilirubin more than three times the upper limit of normal reference value, creatinine (Cr) of at least 3 mg/dL or estimated glomerular filtration rate of not more than 60 mL/min·1.73 m^2^), patients with severe primary diseases of the hematopoietic system, or patients with mental diseases.
7. Patients with malignant tumors.
8. Patients with an allergic constitution or allergy to multiple drugs or foods or patients with an allergy to known components of the study drugs.
9. Patients who cannot take care of themselves or cannot receive oral drugs.
10. Patients who have participated in other drug clinical trials within 1 month.
11. Patients with a suspected or known history of alcohol or drug abuse.
12. Pregnant or lactating women or those who plan to be pregnant.
13. Subjects who, in the judgment of the researcher, are not suitable for study observation.

### Randomization process

The randomized program was provided by statistical professionals from the Institute of Basic Research in Clinical Medicine, China Academy of Chinese Medical Sciences. Participants were randomly assigned in a 1:1 ratio through a centrally controlled, computer-generated, simple randomization schedule. To ensure the smooth progression of the study, the method of competing to recruit participants will occur at eight centers. This study design includes placebo-controlled and double-blind methods. All of the experimental drugs will be packed, obeying the principles of a random assignment table, and will be blinded. The blinding code will be duplicated and will be individually sealed and stored at the sponsor organization and drug clinical trial institution. The study drugs are labeled with serial numbers; when the subject’s random number is generated, the corresponding number of drugs will be transported to the destination within 24 h. All participants, care providers, attending physicians, laboratory staff, and biostatisticians will be blinded to treatment assignment until the database has been locked. Randomization was completed in July 2019. The last participant is expected to be enrolled in December 2020.

### Blinding

This study will blind researchers, participants, evaluators, doctors, nurses, and statistical experts. The blinding codes have been placed in a sealed envelope and will be revealed only when adverse events (AEs) occur or at the terminal analysis.

### Interventions

Eligible participants will be allocated to receive TMYX or placebo (for 12 weeks) in addition to standardized Western treatment. The dose of the drugs, including antiplatelets, stains, ACEIs/ARBs, β-blockers, anti-ischemics, must be in keeping with “the guidelines of acute myocardial infarction in participants presenting with ST-segment elevation”, which was formulated by the Chinese Society of Cardiology in 2015. The TMYXP and placebo were produced and packed in a single batch (production batch number: Tongmai Yangxin: 1070649; Placebo: 20190214) by Tianjin Zhongxin Pharmaceutical Group Co., Ltd., Le Ren Tang Pharmaceutical Factory, which has no conflicts of interest relevant to this study. The test results of drug quality were consistent with the Chinese Medicine Standards of the State Food and Drug Administration. The placebo is composed of 5% crude TMYX and 95% starch, which have the same appearance and scent as the active treatment drugs. Participants will take one bag at a time orally twice daily for 12 weeks, and other Chinese medicine will be avoided in the meantime.

### Outcomes

The primary outcome is LVEDVI = left ventricular end-diastolic volume/body surface area (LVEDV/BSA) and will be detected by three-dimensional ultrasound. The combined secondary outcomes include TCM syndrome score, echocardiogram results, 6-minute walk test results, Seattle Angina Questionnaire score, cardiac magnetic resonance imaging (CMRI) results, biological indicators (growth stimulation expressed gene 2 [ST2], galactose-specific lectin 3 [galectin-3], NT-proBNP, IL-1β, CRP, TNF-α, MMP-2, MMP-9, TIMP-1, TGF-β1, and CTGF), dynamic electrocardiogram results, and experiment event rate (hospitalization rates for HF, death rate of cardiovascular disease, or MI).

### Safety assessment

Safety assessment will be based on the incidence of AEs, including clinically significant changes in physical examinations, vital signs (including blood pressure, heart rate, and pulse), and laboratory examinations. Laboratory examinations to be performed include routine blood and urine parameters, liver and kidney profiles, electrolytes, and myocardial enzymes (detailed monitoring schedule in Table 1). Women of childbearing age will be tested for pregnancy by urine examination at enrollment. The National Cancer Institute’s Common Terminology Criteria for Adverse Events (CTCAE) version 5.0 grading system will be used to classify the nature and severity of AEs [8].

**Table 1:** Research flowchart

Item	Research phase			
	Screening/enrollment	Follow-up		
Time	Before enrollment	4 weeks after enrollment	8 weeks after enrollment	12 weeks after enrollment
	V0	V1	V2	V3
Confirm inclusion and exclusion criteria	√			
Signed informed consent	√			
Basic information	√			
Medical history, treatment history, and allergies	√			
Current medications	√			
Retrieve central randomization	√			
**Efficacy evaluation**				
Echocardiogram	√	√		√
Cardiac magnetic resonance imaging		√		√
Biological indicator	√	√	√	√
Dynamic electrocardiogram	√	√	√	√
Traditional Chinese medicine syndrome score	√	√	√	√
6-minute walk test	√	√	√	√
Seattle Angina Questionnaire	√	√	√	√
Experiment event rate		√	√	√
**Safety**				
Blood pressure, heart rate, etc.	√	√	√	√
Routine blood and urine parameters	√	√	√	√
Liver and kidney profile	√	√	√	√
Electrolytes	√	√	√	√
Myocardial enzymes	√	√	√	√
Urine pregnancy test	√			
Electrocardiogram	√	√	√	√
Appointment for next follow-up		√	√	
Study completion status				√
Case report form examination				√

### Follow-up protocol

The follow-up for all participants will be scheduled at 4, 8, and 12 weeks. The primary and secondary outcomes as well as safety will be evaluated at each follow-up. AEs, especially severe AEs, are to be reported to the research group committee and ethics committee within 24 h. If the principal investigator determines that the AEs may be related to the experimental drugs, the blinding code will be opened and the testing of this subject will be terminated at the same time. All of this must be recorded on the case report form (CRF) and noted to the monitors.

### Compliance

Economic compensation (including transportation, food, and communication reimbursement) is necessary for participants at each follow-up appointment. In addition, at least two telephone numbers will be required to be provided by every participant so that they can be contacted at any time. Moreover, attending physicians will be responsible for answering each patient’s questions. In addition, all of the participants will be compensated 100 Chinese yuan.

To assess adherence to medication, the participants will be asked to fill in the T cards truthfully and to return the unused pills and packages. Non-adherence is defined as when more than 20% of the total amount of medication is not taken as required. The reasons for non-adherence will be recorded in detail on the CRF.

### Sample size estimation

Sample size was calculated on the basis of the expected reduction in LVEDVI after treatment for 3 months. A previous study suggested that the LVEDVs of AMI patients within 24–72 h are approximately 162 ± 57 mL and 162 ± 57 mL at 6 months [9]. According to the pilot experiment of TMYX, the LVEDVI may be reduced by 4 mL/m^2^ within 3 months. Given that the LVEDVI will fall 4 mL/m^2^ following treatment with TMYX added and given a type I error rate of α of 0.05 and power (1 − β) of 0.90, all of the subjects will be assigned in a 1:1 ratio to the treatment group or control group. There should be 100 participants for each arm. Given a dropout rate of less than 20%, a total of 240 participants will be needed to be allocated for the efficacy analysis.

### Statistical approach

Statistical analysis will be performed by using SPSS 21.0 by third-party companies that have no access to the blinding codes, and we will not perform any subgroup analyses. If the primary outcome measure has superior efficacy in one group over the other, a confidence interval will be used for analysis. The secondary end point will be analyzed by a two-tailed test, and significance level will be α of 0.05 (otherwise stated). Continuous variables will be described by the mean and standard deviation. Categorical data will be described by the percentage, frequency, and composition ratio. All effectiveness and safety analyses will be strictly conducted in accordance with the intention-to-treat principle, the full analysis set (FAS) population, the per protocol set (PPS) population, and the safety set (SS) population. A *P* value of less than 0.05 will be considered statistically significant [10].

### Data management and monitoring

During the study, all eight organizations will be monitored bimonthly by the clinical research associate, which has no conflict of interest with the sponsors or researchers. All of the data from the eight organizations will be entered into the clinical data management system by two research assistants. Several measures, including the validation of values, referential data rules, range checks, and consistency checks against data already stored in the database, will be taken to ensure data integrity. Missing or incorrect data will be detected by software programs. All of the modifications of written documentation will be checked by electronic logs and audit trials. Original CRFs will be maintained for five years after the completion of the study. All of the authors will share their raw data and deposit it in the Electronic Data Capture repository (website: http://39.97.182.70:8082/tmyxw/).

## Discussion

Cardiac remodeling, which is the basic mechanism of the occurrence and development of HF, also determines the cardiac function and prognosis of AMI. Prevention and reverse cardiac remodeling play an important role in the treatment of MI.

Cardiac remodeling following MI occurs in both infarct and non-infarct areas, and the major processes include inflammation, cell death, myocardial stunning, hibernating myocardium, myocardial fibrosis of diffused foci or of the whole heart, and scar formation. Remodeling of the infarct area occurs mainly at the early stage of MI and eventually forms scars that are composed mainly of collagen. The remodeling of the non-infarct area usually occurs at the later stage of MI, and cell hypertrophy and fibroblast proliferation are the earliest pathological manifestations. Over time, abundant secretion and deposition of collagen, myocardial compensatory hypertrophy, myocardial interstitial fibrosis, myocardium stiffness, and the enlargement of the cardiac chamber result in progressive loss of ventricular function [11].

TMYX, an effective patent medicine that is commonly used to treat CHD, arrhythmias, and HF, has bright clinical application prospects. With regard to the different pathophysiological factors of cardiac remodeling, previous studies of TMYX have indicated that TMYX can inhibit cardiac hypertrophy; decrease the apoptosis rate of myocardial cells; decrease anti-inflammatory, antioxidant, antiarrhythmic, and myocardial protection; promote angiogenesis; and ameliorate cardiac energy metabolism in a rat model of MI and ischemic reperfusion injury [12–14]. Currently, cardiac ultrasound (CUS) [5, 15, 16] and CMRI [5, 17] are the major tools for the evaluation of ventricular remodeling and myocardial fibrosis at the international level. Above that, some biological indicators can also reflect the progress of fibrosis, such as ST2 and galectin-3 [5, 18–20]. In this trial, patients who have AMI and who receive PCI within 10 days will be the subjects, and the evaluation methods will include CUS; the T1 mapping sequence, cine sequence, and late gadolinium enhancement (LGE) sequence of CMRI; and measurements of biological indicators that reflect the progress of fibrosis. In other words, this trial is designed to demonstrate that the addition of TMYX to conventional treatment will intervene in the development of cardiac remodeling and cardiac dysfunction.

## Trial status

The kickoff meeting of the program was held in Zhengzhou, Henan Province, China, on April 21, 2019. The first participants were recruited in August 2019. All of the participants are scheduled to be enrolled by December 2020. The final amendments (version 2019-V-1.2) occurred on May 22, 2019.

## Abbreviations

CHD: Coronary heart disease
PCI: Percutaneous coronary intervention
AMI: Acute myocardial infarction
HF: Heart failure
CRF: Case record form
TMYX: Tongmai Yangxin pill
CUS: Cardiac ultrasound
CMRI: Cardiac magnetic resonance imaging
ST2: Growth stimulation expressed gene 2
Galectin-3: Galactose-specific lectin 3
MI: Myocardial infarction
LVEDVI: Left ventricular end-diastolic volume index
LVEDV: Left ventricular end-diastolic volume
TCM: Traditional Chinese medicine
ACEI/ARB: Angiotensin-converting enzyme inhibitor/angiotensin II receptor antagonist
AE: Adverse event
